# Pulmonary Surfactant Preserves Viability of Alveolar Type II Cells Exposed to Polymyxin B *In Vitro*

**DOI:** 10.1371/journal.pone.0062105

**Published:** 2013-04-19

**Authors:** Guido Stichtenoth, Egbert Herting, Mario Rüdiger, Andreas Wemhöner

**Affiliations:** 1 Department of Pediatrics, University of Luebeck, Luebeck, Germany; 2 Department of Pediatric Intensive Care and Neonatology, Technical University Dresden, Dresden, Germany; The Ohio State Unversity, United States of America

## Abstract

**Background:**

Exogenous surfactant derived from animal lungs is applied for treatment of surfactant deficiency. By means of its rapid spreading properties, it could transport pharmaceutical agents to the terminal air spaces. The antimicrobial peptide Polymyxin B (PxB) is used as a topical antibiotic for inhalation therapy. Whereas it has been shown that PxB mixed with surfactant is not inhibiting surface activity while antimicrobiotic activity is preserved, little is known concerning the effects on synthesis of endogenous surfactant in alveolar type II cells (ATIIC).

**Objective:**

To investigate ATIIC viability and surfactant-exocytosis depending on PxB and/or surfactant exposure.

**Methods:**

ATIIC were isolated from rat lungs as previously described and were cultivated for 48 h. After incubation for a period of 1–5 h with either PxB (0.05 or 0.1 mg/ml), modified porcine surfactant (5 or 10 mg/ml) or mixtures of both, viability and exocytosis (spontanously and after stimulation) were determined by fluorescence staining of intracellular surfactant.

**Results:**

PxB 0.1 mg/ml, but not porcine surfactant or porcine surfactant plus PxB reduces ATIIC-viability. Only PxB alone, but not in combination with porcine surfactant, rapidly reduces fluorescence in ATIIC at maximum within 3 h, indicating stimulation of exocytosis. Subsequent ionomycin-stimulation does not further increase exocytosis of PxB incubated ATIIC. In presence of surfactant, stimulating effects of PxB and ionomycin on exocytosis are reduced.

**Conclusion:**

PxB alone shows negative effects on ATIIC, which are counterbalanced in mixtures with surfactant. So far, our studies found no results discouraging the concept of a combined treatment with PxB and surfactant mixtures.

## Introduction

Treatment with exogenous surfactant (SF) is mainly applied in primary SF deficiency of the preterm neonate. Occasionally, it is used in conditions of acute lung injury, aspiration of meconium or gastric content and acute respiratory distress syndrome (ARDS), since impairment of endogenous SF synthesis in alveolar type II cells (ATIIC) has been described in these diseases.

SF shows rapid spreading properties and may be used to re-open atelectatic airways. Thus, it rapidly can reach the majority of terminal airspaces. Based upon this knowledge, several authors have claimed a novel role of SF, which is, to transport pharmaceutical agents to the terminal airspaces. Thus, specific treatment of pulmonary disease could be facilitated. However, this requires exclusion of mutual interaction of SF and the transported agent.

The cationic cyclic antimicrobial peptide Polymyxin B (PxB) is an antibiotic isolated from *Bacillus polymyxae*, mainly used for topical treatment of Gram-negative infections. A recent interest is emerging to use it in the context of multidrug resistant Gram-negative infections [1]. PxB doesn’t affect biophysical activity and, moreover, improves resistance of modified porcine SF to meconium *in vitro*, while antimicrobial function is maintained [2]. The bactericidal properties of PxB/SF mixtures are preserved in an animal model and reduced translocation of e.g. *E.coli* from the alveolar compartment to the bloodstream has been described by our group [3]. Thus, PxB is a potential antimicrobiotic additive to SF for treatment or prophylaxis of Gram-negative pulmonary infections.

However, concerns exist in regard to systemic or topic adverse effects of PxB. On the basis of past experience originating from the 1970^th^ and 80^th^, neurotoxic and nephrotoxic properties have been critically re-evaluated recently and may be less than formerly assumed [4].

The objective of this study is to investigate, whether viability or function of ATIIC are impaired by PxB/SF mixtures.

## Materials and Methods

### Ethical Statement

Studies were approved by the local ethical committee of the Technical University Dresden (reference: 24-9168.24-1/2009-13).

### Test Samples

Curosurf (Chiesi farmaceutici S.p.A., Parma, Italy; 80 mg/ml) is a modified natural SF produced from minced porcine lungs. It is in clinical use for treatment of surfactant deficiency and contains about 80 mg/ml phospholipids and 1–2% SF proteins B and C. PxB (Polymyxin B sulphate salt, 8100 units/mg, Sigma-Aldrich, Schnelldorf, Germany) is diluted in saline to a stock solution (10 mg/ml). The following concentrations were used for subsequent testing:

SF (5 and 10 mg/ml),

PxB (0.05 and 0.1 mg/ml),

SF/PxB (5 mg/ml/0.05 mg/ml; 10 mg/ml/0.05 mg/ml or 10 mg/ml/0.1 mg/ml).

### Preparation of ATIIC

ATIIC are isolated from adult Sprague-Dawley rat-lungs as previously described [5]–[7]. In short, lungs of anaesthesized animals are prepared exsanguinous by perfusion and removed from the thorax. After instillation with elastase plus trypsin and stopping the reaction with fetal calf serum, the lungs are minced in DNAse-solution (Deoxyribonuclease I from bovine pancreas, 150KU, Sigma Aldrich, Schnelldorf, Germany) and ATIIC are harvested after serial filtration and centrifugation steps. Macrophages are removed by panning the cells on IgG-coated dishes at 37°C. ATIIC are seeded in 96 well microplates at a concentration of 500,000/well, cultured in Dulbecco’s modified Eagle’s medium (DMEM) plus 10% fetal calf serum, 100 U/L penicillin, 100 µg/ml streptomycin and 24 mM NaHCO_3_ and cultured in 95% humidified air, 5% CO_2_ at 37°C for 48 h. The prepared cells from 3 animals are equally distributed to the experiments defined by test samples and incubation period. Within an identical experiment, 9 wells with cells originating from 3 animals are investigated. After 48 h, the plates are washed twice with DMEM to remove non-adherent cells and then incubated with test samples 1, 3 or 5 h before staining for viability or exocytosis. Only serum free DMEM-incubated cells are used as controls. Finally, the cells are washed to remove test samples.

### Viability

Viability is measured by a previously described fluorescent dye assay [8]; [9]. Cells are incubated with the blue dye resazurin, which is reduced by viable cells to the fluorescent dye resofurin. Fluorescence is measured at 560 nm (excitation) and 590 (emission) in a fluorescence reader (Infinite M200; Tecan; Grödig, Austria).

### SF Exocytosis

After incubation with test samples and washout, cells are stained using lyso tracker green (LTG). Since the fluorescent dye is specifically staining the lipophilic compartments of the ATIIC which is achieved by an active transport into an acidophilic compartment, this process is only performable by vital ATIIC. After 30 min, the dye is removed by a further washout. SF exocytosis is measured indirectly, by determination of the remaining fluorescence of the adhered ATIIC at the bottom of the wells using the fluorescence reader. This method has been described in detail [10]. First, the initial fluorescence is determined and subsequently half of the wells are allocated to stimulation experiments by addition of ionomycin (Sigma Aldrich, Schnelldorf, Germany) at a concentration of 15 µM/well during a period of 30 min at 37°C. Following ionomycin washout, kinetics of exocytosis is followed during 45 min sampling fluorescence during 15 cycles at 3 min each. Thus, spontaneous exocytosis can be compared to ionomycin-stimulated exocytosis.

### Data Analysis

Raw data of fluorescent reader were back-ground corrected, i.e. readings from wells containing non-stained cells were subtracted from raw data. Corresponding raw data of 8–9 wells incubated with identical samples and periods are statistically compared with regard to incubation period or sample. DMEM controls are used as reference. Graph Pad Prism 4.1 software (LaJolla, CA, USA) was used for statistical calculations.

## Results

### ATIIC Viability

Resofurin-fluorescence of ATIIC is significantly decreased by PxB (0.1 mg/ml) after 5 h of incubation, indicating PxB-reduced viability. The effect of PxB is prevented in the presence of surfactant. No significant difference in viability is found in cells exposed to DMEM, SF or PxB (0.05 mg/ml) (Table 1).

**Table 1:** Viability of alveolar type II cells exposed to surfactant and/or Polymyxin B.

Sample	Fluorescence [U]	
	1 h	5 h
**DMEM**	8342±2811	7271±3423
**SF 5 mg/ml**	8703±3403	8627±3809
**SF 10 mg/ml**	8977±4003	8172±3725
**PxB 0.05 mg/ml**	8375±2574	8056±2848
**PxB 0.1 mg/ml**	7890±2743	6162±2206**
**SF 5 mg/ml PxB 0.05 mg/ml**	8623±3466	8737±3547
**SF 10 mg/ml PxB 0.1 mg/ml**	9271±3245	8305±2750
**SF 10 mg/ml PxB 0.05 mg/ml**	9505±3003	8933±2755

Viability of alveolar type II cells (ATIIC) incubated for 1 h or 5 h with modified porcine surfactant (SF) and/or Polymyxin B (PxB) or DMEM control expressed by resofurin-fluorescence, in which viability is decreased in non-proliferating ATIIC (mean±SD; n = 9). A significant reduction in viability is found in ATIIC incubated with PxB (0.1 mg/ml), but not in cells incubated with SF or a mixture of SF plus PxB. No significant reduction of fluorescence is found in any sample comparing 1 h vs. 3 h.

** p<0.01 vs. 1 h (Friedman test of non-Gaussian distributed matched observations.).

### Non-stimulated Exocytosis after Incubation

**Figure 1:**
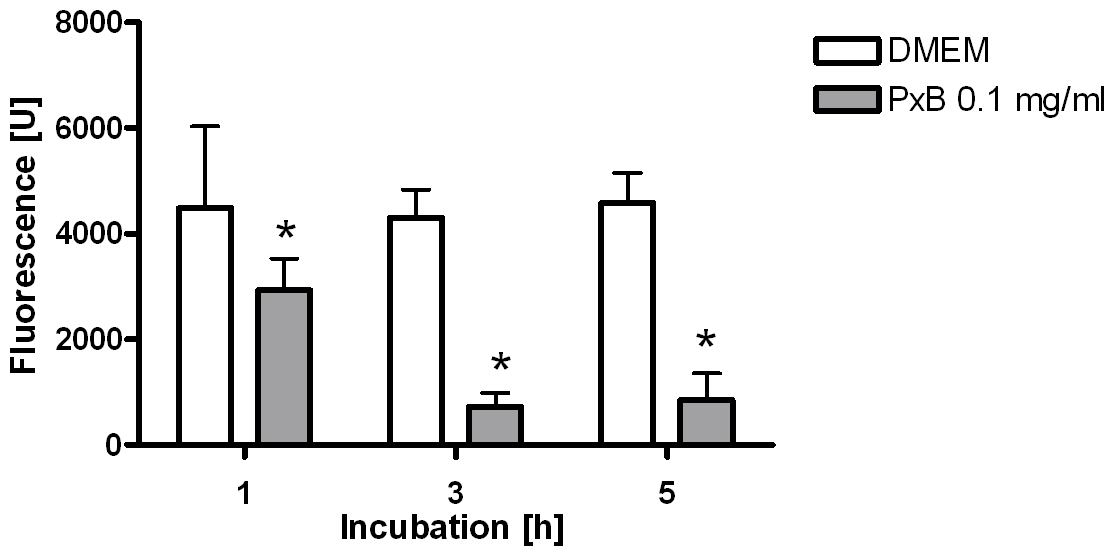
Polymyxin B induces exocytosis of alveolar type II cells. Initial lyso tracker green fluorescence of alveolar type II cells 30 min after staining, comparing cells incubated with Polymyxin B (PxB) to controls (DMEM). The fluorescence in cells exposed to PxB decreases in a time-dependent manner, whereas it remains stable in controls. Bars are mean+SD, n = 14–16. *: p<0.01 vs. DMEM.

**Figure 2:**
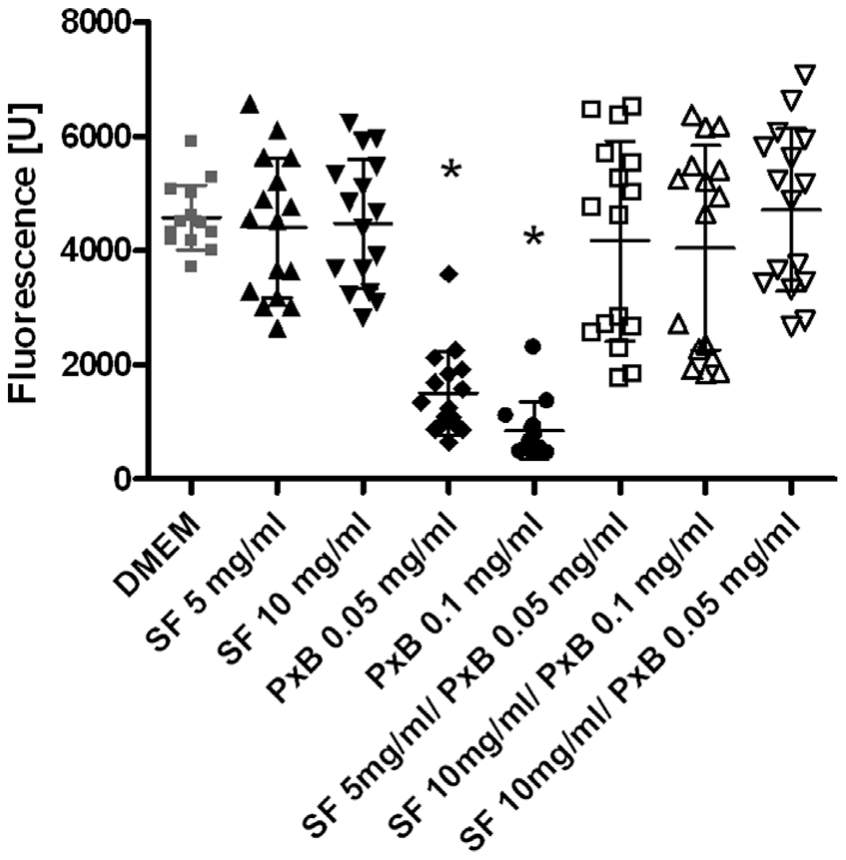
Pulmonary surfactant prevents exocytosis that is induced by PolymyxinB. Fluorescence (lyso tracker green) scattergram of alveolar type II cells after 5 h incubation with mixtures of modified porcine surfactant (SF) and/or Polymyxin B (PxB), 30 min after staining of lamellar bodies, compared to controls (DMEM). Fluorescence in cells exposed to PxB is significantly reduced compared to DMEM, SF or in mixture with SF. Bars show mean ± SD, n = 14–16. *: p<0.01 vs. DMEM, SF and SF plus PxB.

LTG-fluorescence of ATIIC incubated with PxB (0.1 mg/ml) decreases in a time-dependent manner, indicating increased exocytosis compared to DMEM-incubated cells, in which LTG-fluorescence remains stable (Fig. 1). After 5 h, fluorescence of cells exposed to PxB 0.05 and 0.1 mg/ml is significantly lower compared to DMEM controls, SF or mixtures of SF and PxB (p<0.01; Fig. 2).

### Stimulated Exocytosis

**Figure 3:**
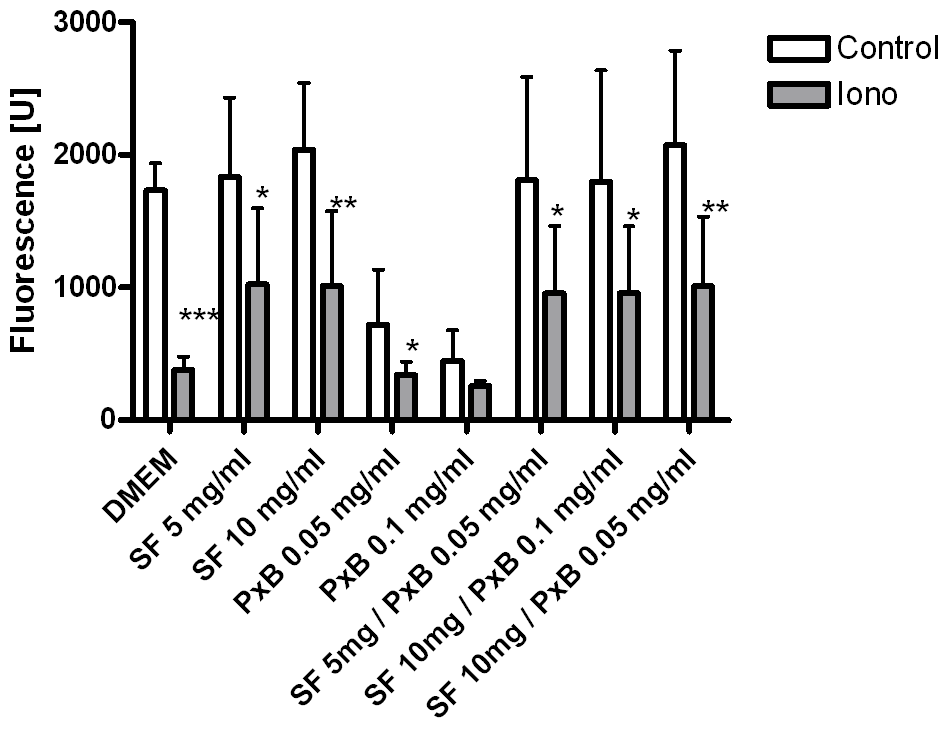
Ionomycin doesn’t further increase exocytosis wich is induced by Polymyxin B. Ionomycin-stimulated exocytosis (Iono) compared to non-stimulation (control): Fluorescence of alveolar type II cells incubated 5 h with medium (DMEM) or different mixtures of modified porcine surfactant (SF) and/or Polymyxin B (PxB). Bars show mean ± SD, n = 7–8. *: p<0.05; **:p<0.01; ***:p<0.001 vs. control (t-test).

Ionomycin-stimulation is used to induce maximum exocytosis of ATIIC. Fig. 3 shows ionomycin-stimulated exocytosis of ATIIC incubated for 5 h with test samples in comparison to non-stimulated cells. The highest increase in exocytosis corresponding to maximum reduction of fluorescence is found in ionomycin stimulated ATIIC exposed to DMEM (p<0.001). However, after incubation with PxB 0.1 mg/ml, a subsequent stimulation with ionomycin doesn’t have a significant effect on exocytosis. Thus, ionomycin doesn’t cause a further increase in exocytosis in PxB-incubated cells. After ionomycin stimulation, all cells incubated with SF-containing test samples show a LTG-fluorescence of ∼1000 U, which is increased compared to DMEM incubated cells, suggesting a lower exocytosis of endogenous SF in presence of exogenous SF (Fig. 3).

## Discussion

In presence of Curosurf (5 or 10 mg/ml), viability of ATIIC is stable for the incubation period of 5 h. This is confirmed by an investigation comparing the effects of Curosurf 10 mg/ml on viability of rat ATIIC, mouse ATIIC and the adenocarcinomia human alveolar basal epithelial cells [11]. We found that viability of ATIIC is significantly reduced by 5 h incubation with pure PxB at 0.1 mg/ml. This effect is counterbalanced, if cells are incubated with PxB in presence of SF. PxB induces maximum exocytosis of ATIIC within 5 h. This effect cannot be further increased by subsequent ionomycin-stimulation. Ionomycin- and PxB-stimulated exocytosis are reduced by SF. We found an effect of PxB 0.1 mg/ml, but not by PxB 0.05 mg/ml or in the presence of SF. The ratios of SF/PxB chosen in the experiments correspond to the ratio of the recommended doses of Curosurf (100–200 mg/kg) and of aerosolized PxB (2.5 mg/kg/d) [12]. This ratio has already been used during evaluation of biophysical and physiological properties of Curosurf/PxB [13] and showed an efficient activity against *E.coli in vitro* [2]. In the latter study and in a formulation of PxB encapsulated in dipalmitoylphosphatidylcholine : cholesterol a sustained PxB-release from the lipophilic compartment was suggested [14].

So far, signaling pathways of PxB on ATIIC are unknown. In contrast, it has been shown that PxB has modulating effects on different cell types and their functions. PxB induces partial maturation of human dendritic cells [15]. Ferrari and colleagues found *in vitro* a modulation of a pore forming receptor in human embryonic kidney cells induced by PxB [16]. Moreover, PxB causes modulation of the purinergic receptor P2X7 [17]. Although surfactant secretion may be induced via P2X7 receptors of alveolar type I cells via paracrine cell-cell-communication, this receptor type is exclusively present on ATIC, but not on ATIIC [18]. Recently, additional purinergic receptors on ATIIC like P2X4 have been found [19]. At present, the direct mechanism by which PxB induces surfactant secretion in isolated ATIIC is unclear. This should be a subject of future research.

PxB is a cross-linking peptide and directly interacts with surface films enabling a link between two phospholipid monolayers. Thus, release of PxB from the lipo-protein-complex of SF may be delayed, reducing peak concentrations, which could be an explanation for reduced cell toxicity of SF/PxB mixtures. Studies on liposomal preparations of PxB suggest that a liposomal environment may be protective for polycationic antibacterial agents like PxB in extending steady drug concentrations after topic instillation into the lungs [20]. This is supported by our previous observation, that the growth of *E.coli* in meconium is slightly more reduced by PxB alone compared to PxB in presence of SF. SF might be more useful as a vehicle for PxB as rapid spreading is maintained in the mixture and, compared to liposomal preparations, SF/PxB is able to re-open atelectatic airways [2]. Thus, this combination might be applied in the clinical condition of lung injury. In the 1970s adverse effects like nephrotoxicity and neurotoxicity caused that the clinical use of polymyxins as systemic antibiotics was abandoned [21]. Nowadays, a renaissance of polymyxins is discussed as supported by clinical data that are not confirming major adverse effects [17] and moreover, efforts are made to synthesize new polymyxin derivates [22].

To study viability and SF-exocytosis, multi well plate assays were used, that have been described in detail and are considered as stable, reproducible and reliable [7]. However, some methodological aspects have to be discussed for the present study. The LTG-fluorescence of cells incubated with PxB is reduced by 35% after 1 h and by 81% after 5 h compared to DMEM, as indicated by Fig. 1 and 2. In parallel, viability of the cells is reduced by ∼8% after 3 h and by only ∼22% after 5 h: Hence, the possibility of insufficient staining of PxB incubated ATIIC may be discussed, as the uptake of the dye possibly is delayed or exocytosis may be accelerated. The fact that PxB is a polycationic agent and that the labelling of lamellar bodies depends on protonation [7] could suggest an interaction of PxB and LTG staining. However, the design of the study implies that only viable cells with a satisfactory energy status adhere at the bottom of the plates. Viability and sufficient energy status are simultaneously intracellular requirements enabling LTG staining. In addition, the vast bulk of PxB should have been washed out before staining with LTG starts.

In conclusion, our data suggest a moderate reduction of ATIIC viability induced by incubation with PxB which may be counterbalanced by co-incubation with SF. Moreover, incubation with pure PxB stimulates spontaneous SF exocytosis. Interestingly, no negative effects of SF/PxB-mixtures on vitality and SF-exocytosis are found. Thus, the data encourage our intention to use SF as a vector for PxB administration, e.g. in treatment of pulmonary Gram-negative bacterial infections.
